# Gabapentin-Induced Urinary Incontinence: A Rare Side Effect in Patients with Neuropathic Pain

**DOI:** 10.1155/2015/341573

**Published:** 2015-08-16

**Authors:** Sibel Kibar, Sibel Demir, Nebahat Sezer, Belma Füsun Köseoğlu, Meltem Dalyan Aras, Bilge Kesikburun

**Affiliations:** Department of Physical Medicine and Rehabilitation, Ankara Physical Medicine and Rehabilitation Education and Research Hospital, Ankara, Turkey

## Abstract

Gabapentin is a first-line agent for neuropathic pain management and has a favorable safety profile. The literature includes a few cases of gabapentin-induced incontinence, and most of them involved patients with epilepsy who were between the ages of 12 and 43 years. Herein, we present three patients with neuropathic pain due to different diagnoses, and, to our knowledge, these are the oldest reported cases of urinary incontinence caused by gabapentin therapy. A 56-year-old female patient who underwent hip arthroplasty developed a sciatic nerve injury and neuropathic pain postoperatively. Ten days after she began taking gabapentin to relieve her pain, she experienced daily urinary incontinence. In another instance, a 63-year-old female patient was diagnosed with complex regional pain syndrome, and seven days after the initiation of gabapentin therapy, urinary incontinence developed. In addition, a 66-year-old male patient with neuropathic pain due to cervical disc pathology complained of urinary incontinence after the onset of gabapentin therapy. After discontinuing this drug, the incontinence symptoms resolved in these patients on the seventh, the first, and the second days, respectively. Physicians who administer gabapentin should inform their patients about the potential risk of gabapentin-induced incontinence and its negative impact on quality of life.

## 1. Introduction

Gabapentin is a structural analogue of gamma aminobutyric acid (GABA) and was originally designed as an anticonvulsant [1]. Subsequently, gabapentin became a first-line agent for neuropathic pain management [2]. Gabapentin primarily exerts its effect via selectively interacting with the *α*2*δ*-1 subunit of voltage-dependent calcium channels [2]; however, its precise mechanism of action remains unclear [3], as its effects involve numerous targets requiring further research [2]. Additionally, gabapentin is widely used not only to treat seizures and neuropathic pain but for numerous other indications, including anxiety and sleep disorders, because of its apparent lack of toxicity [4]. Nevertheless, few studies have reported incontinence associated with the use of gabapentin in patients with epilepsy and neuropathic pain [5–7]. Herein, we present 3 patients with urinary incontinence caused by gabapentin therapy for neuropathic pain.

## 2. Case Reports

### 2.1. Patient 1

A 56-year-old female underwent hip arthroplasty to relieve hip pain caused by coxarthrosis. Postoperatively, a reduction in hip pain was noted, but the patient complained of right leg paresthesia and ankle weakness. Manual muscle testing of the right leg showed that ankle dorsiflexion was 4/5 and toe dorsiflexion was 3/5. There were L4-L5-S1 paresthesia and allodynia. ENMG findings indicated sciatic nerve injury. Gabapentin 400 mg/day p.o. was started and progressively increased to 2400 mg/day. Following administration of gabapentin, the patient's paresthesia was greatly reduced; however, 10 days after starting the therapy, she experienced daily urge urinary incontinence. Incontinence was mix type urinary incontinence. Neurological and systemic physical examination, laboratory tests, and urinalysis were normal. Lumbosacral spine magnetic resonance imaging (MRI) showed no abnormality. The vaginal parity of the patient was 4. Upon reviewing the literature, we considered that gabapentin was the cause of incontinence and discontinued the drug. Her symptoms resolved 7 days after discontinuation of gabapentin and the patient remained continent during 11 months of follow-up.

### 2.2. Patient 2

A 63-year-old female previously diagnosed with complex regional pain syndrome presented with left hemiplegia. Gabapentin 400 mg/day was initiated and the dose was increased every 3 days to 2400 mg/day. The patient reported satisfactory pain relief with gabapentin; however, 7 days after starting the maximum dose of gabapentin (2400 mg/day), she developed urge urinary incontinence.

Cranial MRI did not show any pathology. Laboratory tests and urinalysis were normal. The vaginal parity of the patient was 6. Gabapentin was administered at 400 mg for 3 days and then discontinued; the patient's incontinence resolved 5 days later and she remained continent during 1 year of follow-up.

### 2.3. Patient 3

A 66-year-old male presented with left arm paresthesia due to spinal stenosis and a herniated disc at cervical vertebrae C5-6 and C6-7. Gabapentin 400 mg daily was started and on the following day the patient complained of urinary incontinence. MRI of the cervical spine did not show any new pathology, and laboratory tests and urinary analysis were in the normal range. On the day following the cessation of gabapentin, the patient's incontinence resolved.

## 3. Discussion

To the best of our knowledge, the literature includes just 5 cases of gabapentin-induced incontinence, 3 of which involved both rectal and urinary incontinence and 2 involved only urinary incontinence. In all cases, incontinence occurred after 1–4 weeks of gabapentin treatment at dosages of 600–3600 mg/day. Among the 5 reported cases, 4 had epilepsy and were treated with gabapentin as adjunctive therapy [5, 7]; the fifth patient had tumor-related neuropathic pain [6]. Herein, we reported 3 patients with neuropathic pain due to different pathologies that developed gabapentin-induced incontinence. Incontinence occurred after 7–10 days of gabapentin 2400 mg/day in 2 of the patients. Incontinence persisted as long as the patients continued to use gabapentin and stopped soon after (5–7 days) it was withdrawn, as in the previously reported cases. The other presented patient complained of urinary incontinence only 1 day after starting gabapentin 400 mg/day and had a very short recovery period (1 day). The age range of the 5 previously reported cases was 12–43 years, versus 55–66 years in the 3 presented patients; as such, we think they are the oldest reported cases of gabapentin-induced incontinence.

According to Gil-Nagel et al., gabapentin-induced incontinence was thought to be associated with preexisting frontal lobe damage, because of their patients' primary diagnosis of epilepsy with frontal lobe damage [5]. Gabapentin does not bind to plasma proteins and is thus distributed in most organs and tissues. Therefore, the mechanism accounting for incontinence could involve not only the brain and the spinal cord but also the local effects of gabapentin on gastrointestinal tract and urinary tract [5]. Iyer et al. proposed a different gabapentin-induced incontinence mechanism based on the relationship between gabapentin and afferent C-fiber nerve activity [6]. Myelinated A-*δ* and unmyelinated C-fibers are the most important afferents that initiate micturition and pass through the pelvic nerves to the sacral spinal cord [8].

A-*δ* and C-fibers both have alpha-2-delta subunit Ca^++^ channels. Submucosal receptors of bladder stimulate presynaptic transmitter release mediated by Ca channels, which results in detrusor contraction [9]. Therefore, upregulation of bladder C-fiber afferent nerve function may also play a role in certain cases of urge incontinence and overactive bladder [10]. Gabapentin has high affinity to alpha-2-delta subunit Ca^++^ channels which can reduce the calcium current type L [11]. The detrusor contraction decreases with the declination of calcium current. According to Antonio et al., gabapentin decreases the detrusor hyperactivity by reducing the afferent signaling of the C- and A-*δ* fibers responsible for filling sensation [12].

Decreased detrusor hyperactivity may increase maximal capacity and incontinence. The vaginal parity of two female patients is 4 and 6, respectively. Vaginal parity is mostly associated with stress urinary incontinence but it is also associated with all types of incontinence [13]. Weak external urethral sphincter developed by multiple vaginal parity may support this incontinence for our female patients. Vaginal multiparity of these patients might induce the relaxation of the external sphincter. Incontinence symptoms of the third patient were relieved in one day. The reason behind this short relief time might be the male gender.

It was reported that tonic GABAergic inhibitory control of micturition is mediated by GABAs A and B [9]. Although the general opinion in the literature is that gabapentin does not act on GABA receptors, this subject remains contentious. Some studies reported that GABA B receptors play a role in gabapentin's mechanism of action [14]. It was suggested by Cheng and Chiou that GABA B receptor activation is involved in the therapeutic actions of gabapentin other than analgesia [15]. During micturition, the external striated sphincter relaxes due to inhibitory modulation of GABA neurons [16]. GABA B receptors have a minor effect on normal relaxation of the striated urethral sphincter via motoneuron excitability [17]; thereby, we think that in some patients gabapentin may cause incontinence by overrelaxation of the external sphincter via excessive influence of GABA B.

## 4. Conclusion

It is thought that GABA B receptors have a minor effect on normal relaxation of the striated urethral sphincter and as such we think that in some patients gabapentin may cause incontinence via over relaxation of the external sphincter via excessive influence of GABA B. Physicians administering gabapentin should inform patients about the potential for gabapentin-induced incontinence.
